# Heavy metals concentrations and speciation of Pb and Ni in airborne particulate matter over two residential sites in Greater Cairo – reflection from synchrotron radiation

**DOI:** 10.1107/S1600577522003058

**Published:** 2022-04-22

**Authors:** Mohamed H. E. Monged, N. G. Imam, Giuliana Aquilanti, Simone Pollastri, A. M. Rashad, János Osán

**Affiliations:** a Department of Siting and Environment, Nuclear and Radiological Safety Research Center (NRSRC), Egyptian Atomic Energy Authority (EAEA), Cairo 11762, Egypt; b Experimental Nuclear Physics Department (Solid State Laboratory), Nuclear Research Center (NRC), Egyptian Atomic Energy Authority (EAEA), Cairo 13759, Egypt; c Elettra Sincrotrone Trieste, Strada Statale 14, km 163.5 in AREA Science Park, Basovizza, Trieste 34149, Italy; d Accelerators and Ion Sources Department, Nuclear Research Center (NRC), Egyptian Atomic Energy Authority (EAEA), PO 13759, Abu Zaabal, Cairo 11762, Egypt; e Central Laboratory for Elemental and Isotopic Analysis, Nuclear Research Center (NRC), Egyptian Atomic Energy Authority (EAEA), PO 13759, Abu Zaabal, Cairo, Egypt; f Centre for Energy Research, Konkoly Thege M. út 29–33, 1121 Budapest, Hungary

**Keywords:** synchrotron radiation, XANES, particulate matter, heavy metals, Pb speciation, Ni speciation, risk assessment, X-ray fluorescence, inductively coupled plasma-mass spectrometry, carcinogenic risks

## Abstract

Synchrotron radiation-based techniques [X-ray absorption near-edge structure (XANES) and X-ray fluorescence (XRF)] combined with inductively coupled plasma-mass spectrometry (ICP-MS) were used for the assessment of heavy metals concentrations as well as lead (Pb) and nickel (Ni) speciation in airborne particulate matter (PM_10_) over two residential sites in Greater Cairo. Nineteen 24 h high-volume samples collected at Giza (G) Square and Helwan (H) University (Egypt) were selected for this study. Mean concentrations of heavy metals in PM_10_ at both sites were found to have the same descending order of Pb > Cu > Ni > Cd > Co > As, of which concentrations of Pb, Cu, Ni and Cd in H samples were higher than those in G samples. For Pb, synchrotron-based XRF results were in good agreement with concentrations obtained by ICP-MS. The XANES spectra of PM_10_ at the Pb *L*_2_-edge and Ni *K*-edge were compared with those of Pb and Ni in model standard compounds to provide information on the potential oxidation states as well as the chemical forms of those elements. The data show that Pb has similar chemical environments in both series G and H with the predominance of Pb^2+^ oxidation state. Nickel was found as Ni(OH)_2_, NiO and Ni metal in the analyzed samples. However, the content of Ni in the background filter shows a very strong interference with that of the collected PM_10_. Carcinogenic and non-carcinogenic risks resulting from the inhalation of the studied heavy metals were assessed for children and adult residents and were found below the safe limits, at both sites.

## Abstract 2

XANES and X-ray fluorescence combined with inductively coupled plasma-mass spectrometry were used to quantify and identify heavy metals concentrations as well as the chemical speciation of Pb and Ni, which have considerable public health impacts in Egypt.

## 1. Introduction

Particulate matter (PM) is emitted into the atmosphere through both natural and anthropogenic sources. Wind-blown soil dust, sea sprays and volcanic ash comprise natural sources, while combustion processes, industrial and vehicular emissions are among anthropogenic sources. However, the environmental consequences of anthropogenic sources are more significant than those of natural sources. Owing to the substantial increase in the volume of traffic, urbanization, industrialization and population growth in recent decades, the issues associated with particulate emissions have increased considerably (Ahmad *et al.*, 2019 ▸; Carmichael *et al.*, 2009 ▸; Ilyas, 2007 ▸; Hassanien *et al.*, 2009 ▸).

Atmospheric pollution by PM in Greater Cairo (GC) is one of the major pollutants leading to the degradation of its air quality. Resuspended soil dust, mobile source emissions, oil combustion and open burning are the major contributors to PM_10_ (particles with aerodynamic diameter less than 10 µm) levels in the GC area (Lowenthal *et al.*, 2014 ▸). The annual mean concentrations of PM_10_ in GC show that ∼70% of the region is considered unhealthy for the public (200–250 µg m^−3^) (WHO, 2005 ▸, 2018 ▸; Lowenthal *et al.*, 2014 ▸; Higazy *et al.*, 2019 ▸).

Heavy-metal polluted air in Cairo is attributed to multiple emission sources including road vehicles, industrial and manufacturing facilities, and municipal waste sites and incinerators. The number of vehicles has been increasing over recent decades, already exceeding 7.3 million at the end of 2013, of which Cairo and Giza account for 29.5% and 12%, respectively (CAPMAS, 2013 ▸). Industrial zones within GC (Helwan, Shoubra El-Khaima and El-Tebeen) and other different areas with various industrial activities, in addition to small industries, are widely distributed in the populated areas. Moreover, open-air burning of solid municipal waste has been classified as the largest source of suspended particles in the GC air with 36% of the total annual emission. Exhausts of road vehicles account for 26%, whereas industrial emissions and agricultural-waste open burning account for 32% and 6%, respectively (EEPP, 2004 ▸; Hassanien *et al.*, 2009 ▸).

PM can carry both inorganic and organic contaminants through atmospheric processes and tends to be highest in urban locations (Chan *et al.*, 2000 ▸). Ambient particulates are also characterized as carriers of toxic heavy metals into the human body through inhalation into the lungs (Avino *et al.*, 2008 ▸; Gharaibeh *et al.*, 2010 ▸; Oucher *et al.*, 2015 ▸; Hsu *et al.*, 2016 ▸; Ahmad *et al.*, 2019 ▸; Khan *et al.*, 2020 ▸).

Study of the distribution and health effects of heavy metals has drawn considerable interest as being associated with PM from various sources, especially, heavy metals with elevated toxicity and great diffusion in the environment, such as Cd, As, Co, Cr, Hg, Ni and Pb. Heavy metals differ mainly from other toxic substances in the sense that they are neither created nor destroyed by humans. Nevertheless, human utilization influences the potential health effects, either by direct emissions, that is, by human contribution to air, water, soil and food, or by altering the speciation or biochemical form of the element (Beijer & Jernelöv, 1986 ▸).

The chemical speciation of a metal is often one of the most important features determining its toxicity, affecting its absorption, membrane transport and excretion, as well as its toxicity at the cellular or molecular target. For example, inorganic Mn(III) species are generally more toxic than other oxidation states of the element. Likewise, Cr(III) is probably an essential element but Cr(VI) is genotoxic and carcinogenic. In contrast to Cr and Mn, where the higher oxidation states are more toxic, more reduced species of inorganic As are more toxic than higher oxidation states (Templeton, 2015 ▸). Insoluble Ni(II) species, especially sulfidic Ni(II), have a higher carcinogenic risk (CR) than when they are in soluble form (Schaumlöffel, 2012 ▸).

The Egyptian Environmental Affairs Agency (EEAA) has issued and applied some regulations and policies in order to control pollution levels in the GC area. These regulations started by ordering the phase out of the use of leaded gasoline, the relocation of lead smelters outside populated areas, as well as the use of modern smelting technology with lower emission rates. As a result, air quality in GC has considerably improved. As an indicator of such improvement, Pb concentrations in the GC atmosphere have decreased considerably from 7.2 µg m^−3^ (during the summer of 2002) to 0.42 µg m^−3^ (during 2011), which is below the national (EEAA, 2011 ▸) as well as international (WHO, 2000 ▸) air-quality-limit values for Pb (0.5 µg m^−3^).

Synchrotron-based techniques have played, over the last three decades, a significant role in materials and environmental sciences (Zaki & Imam, 2017 ▸). Their ability to gain accurate data at the atomic and molecular level on both local and long-range structures, jointly with the increasing ability to carry out time-resolved studies, has affected our understanding of various key types of functional materials, while their strength as analytical tools has a major influence on issues of environmental studies (Catlow, 2015 ▸).

X-ray absorption near-edge structure (XANES) is ideally suited to provide information about the valence state, and local and electronic structures of elements (Aquilanti *et al.*, 2017 ▸). The high brilliance provided by synchrotron radiation (SR) allows one to study materials where the contaminants (*e.g.* Pb and Ni) are highly diluted (Imam *et al.*, 2019 ▸). Furthermore, the chemical selectivity of XANES allows one to investigate the elements of interest within a matrix (Wang *et al.*, 2007 ▸). In that regard, the standard method used for deducing the valence-states information of the target ions is a fingerprint method based on the comparison of the XANES spectra of the element of interest along with those of the reference compounds.

In this work, inductively coupled plasma-mass spectrometry (ICP-MS) and synchrotron-based X-ray fluorescence spectroscopy (XRF) were used for the assessment of heavy metals associated with atmospheric PM_10_ samples collected from Giza (G) and Helwan (H) in Egypt. Furthermore, the XANES technique was used to perform the chemical speciation of Pb and Ni in order to identify their valence states, as well as their possible chemical forms, as they are metals of public health impact. The studied sites are highly populated areas with a variety of pollution sources. Moreover, Giza and Helwan are located in the south and south east of GC, respectively. Based on the dominant wind direction, Giza and Helwan are affected by emission sources located in the north and northwestern parts of GC. Therefore, it is important to continuously determine the concentrations of PM_10_ and the associated heavy metals in order to evaluate carcinogenic and non-carcinogenic health risks of inhalation of PM_10_ for residents of these sites. It is expected that these work outcomes will help the decision makers and the concerned authorities in evaluating the feasibility of the pollution reduction strategies as well as the residential air quality.

## 2. Materials and methods

### 2.1. Sampling

Nineteen PM_10_ samples were collected from two sampling stations at the center and south east of GC (Egypt), *i.e.* nine samples from Giza (G1 to G9) and ten from Helwan (H1 to H10), respectively. The Helwan sampling station is situated inside Helwan University (29° 52′ 1.87′′N, 31° 18′ 53.84′′E), which is north of a Helwan industrial area that consists of an iron and steel company and three cement factories. In addition to Arab Abu-Saed, which is an industrial area that contains hundreds of red-brick factories and metal-processing workshops, two thermal power plants in Tebeen (south of Helwan) operated by residual fuel mazut (rich in S, Ni, Pb and other heavy metals) are another significant source of air pollution around Helwan.

On the other hand, Giza station is positioned inside Giza Square (30° 0′ 57.06′′N, 31° 12′ 40.33′′E), which is surrounded by heavy-traffic roads and near the train and metro stations. Two mazut-fired thermal power plants are located at Shubra Al-Kheima and Cairo West, ∼15 km north of Giza Square; in addition to the Abu Rawash industrial zone (∼11 km WNW of Giza Square), which is characterized by metal processing and metallurgical industries, such as the car industry, metal-alloy industries, plastic and PVC pipes, chemical industries, *etc*. Furthermore, solid-waste incineration is a common practice in the suburb areas around both sampling stations. Vehicles emissions and road dust are also potential sources of emissions because of the presence of too many arterial roads along the whole GC area.

**Figure 1:**
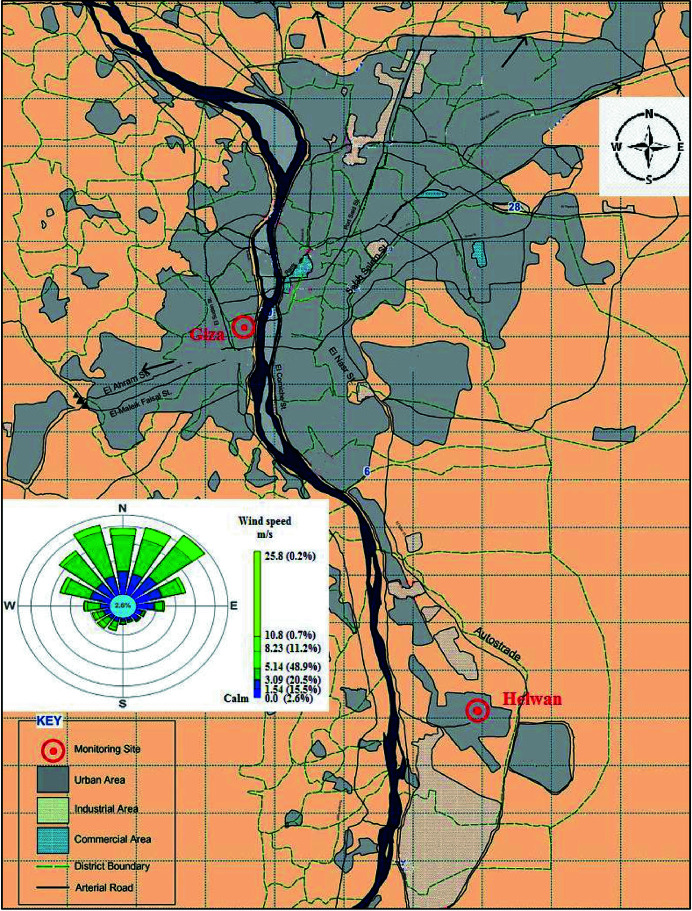
The wind rose of the GC area and the locations of the Giza and Helwan sampling stations (modified from Lowenthal *et al.*, 2014 ▸).

Fig. 1 ▸ shows the locations of the sampling stations and the wind rose (wind speeds and wind directions) of the GC area. According to Fig. 1 ▸, Giza and Helwan are located in the south and south east of GC, respectively, which make them the main receptors of pollutants from pollution sources on the north and northwestern parts of GC according to the wind rose of GC.

### 2.2. Collection of airborne PM (PM_10_) samples

The high-volume sampler (General Metal Works, Model GMW-1200, USA) used for particulate collection was operated at a high flow rate of 1.13 m^3^ min^−1^ (±10%) drawing the air sample into a covered housing and through binder-free Whatman Grade GF/A glass microfiber filters. The mass of the particles collected on the filter was determined by the difference between gravimetric measurements of the filter mass before and after exposure. This method has also been applied for the collection of aerosols used for analysis of aerosol chemical species (Kubilay & Sayda, 1995 ▸; Derek, 1992 ▸). The glass filters used had a particle filtration size of 1.6 µm with 98% collection efficiency. The used filter media had 8 × 10 inch dimensions, 260 µm thicknesses and a basis weight of 53 g m^−2^.

### 2.3. ICP-MS measurement

Filter samples were subjected non-destructively to synchrotron XRF to measure the content of heavy metals of interest. Due to the high blank values of Ni, Zn and Ba, only the peak of Pb was high enough to be detected with a degree of confidence in the XRF spectrum, while the other metals were hindered by peaks of the matrix elements. Therefore, in order to quantify the other heavy metals as well as Pb and Ni, ICP-MS was employed.

First, a piece of fixed area (64 cm^2^) was taken from each sample, as well as from blank filters. Then each piece was weighed to obtain the actual weight of the collected particles. Later, samples and blank filters were digested using concentrated acids: 10 ml of HF (40%), 10 ml of HClO_4_ (70%) and 10 ml of HNO_3_ (65%), and evaporated until dryness. Residues from the last step were digested in 1 ml of concentrated HCl (37%), and then 10 ml of concentrated HNO_3_ were added to each sample and heated until near dryness to convert the matrix into nitrate form. The digested samples were diluted to 15 ml using deionized water and filtered through Whatman #42 filter paper.

The prepared samples were stored at 4°C prior to the quantification of heavy metals. Analyses were performed using a triple quadrupole ICP-MS system (iCAP TQ, Thermo Scientific) installed at the Central Laboratory for Elemental and Isotopic Analysis, Nuclear Research Center, Egypt. The instrument is optimized using an instrument autotune solution. An ICP multi-element standard solution IV (Merck, Darmstadt, Germany) was used for the preparation of the calibration curves, quality control and method validation.

All standards and samples were measured for three runs, with five sweeps for each run. Samples are prepared using double distilled and deionized water (Evoqua Milli-Q purification system 18.2 MΩ) in 1% HNO_3_. Measurements were performed in single quad mode with kinetic energy discrimination, for all elements except As. Measurements of As were performed in triple quad mode, using oxygen as a reaction gas to overcome the problem of interferences resulting from the matrix of the samples under plasma conditions.

For quality control, and to avoid contamination during sample preparation and analysis, acid-washed glassware was used. The glass fiber filters were equilibrated in a desiccator for 24 h after sampling to avoid the impact of relative humidity. An analytical microbalance with sensitivity of 10 µg was used to weigh the filters before and after sampling. Reagent blanks were prepared and simultaneously digested and analyzed along with each batch of collected samples to check the possible contamination of chemicals and glassware. A mixed standard of the measured metals was run after each batch of samples to check the stability of the pre-calibrated ICP-MS system, and variation was satisfactory (<10%).

### 2.4. Synchrotron radiation experiment

XANES spectra were collected at the Ni *K*-edge (8333 eV) and Pb *L*_2_-edge (15 200 eV) at the 11.1 XAFS beamline of Elettra Sincrotrone Trieste, Italy (Di Cicco *et al.*, 2009 ▸). The X-ray beam was monochromated using a double-crystal Si(111) monochromator. Energy calibration was accomplished by collecting XANES spectra of Ni and Pb metal foils and assigning the first inflection point to 8333 eV and 15 200 eV for the Ni *K*-edge and Pb *L*_2_-edge, respectively. The energy stability was monitored by collecting simultaneously both the data of the sample and the data of the reference foils placed in a second experimental chamber after the sample and after the I_1_ ionization chamber. We chose to collect the XANES signal of Pb at the *L*_2_-edge instead of the *L*_3_-edge because of the presence of As, for which the *K*α_1_ line is overlapping with the *L*α_1_ line of Pb (10 543 eV and 10 551 eV, respectively), although the concentration of As is tiny in the probed samples.

The following samples and model compounds were measured:

(i) Giza (G1 to G9) series and Helwan (H1 to H10) series.

(ii) Lead foil, PbO, NiO and Ni(OH)_2_ as Pb- and Ni-bearing reference compounds.

All the spectra of the samples were collected in the fluorescence mode using a silicon drift detector of 80 mm^2^ active area (AXAS-M, Ketek, Munich, Germany), whereas those of model compounds were collected in the transmission mode using the ionization chamber. The XANES spectra of the samples were collected in the energy range 8250–8575 eV for Ni and 15 150–15 295 eV for Pb at room temperature and in air, and mounted on a multisample holder oriented at 45° with respect to the incident beam. A variable step as a function of energy was used: a large step (5 eV) in the first 200 eV of the XANES spectrum and a smaller step (0.2 eV) in the farther XANES region, while a *k*-constant step of 0.03 Å was opted for in the EXAFS region. For each absorption edge on each collected PM_10_ sample and model compound, a minimum of three XANES spectra were collected and merged in order to improve the statistics. The merged XANES spectra were then normalized with respect to the high-energy side of the curve using the *Athena* software (Ravel & Newville, 2005 ▸). The normalized XANES spectra were then analyzed through linear combination fitting (LCF) of the spectra from model compounds, using the *Athena* software. LCF was performed at least three times on each spectrum, trying different normalization strategies, and both on raw and smoothed data, in order to test the reproducibility of the obtained results.

Full XRF spectra were also collected for each sample, with an incident-beam energy of 17 500 eV, to excite most elements present. Fitting of X-ray spectra and quantification were performed using the *PyMCA* software package (Solé *et al.*, 2007 ▸), using the 2.56 wt% Zn content of the GF/A filter as the internal standard. Absorption correction was performed, taking into account that the filters were folded with the glass-fiber-filter layer facing the incoming beam and detector and the 45°/45° geometry. Air concentrations of Pb were calculated after blank subtraction; however, the high blank values of unloaded blank filters hindered the determination of Ni.

### 2.5. Risk assessment of inhalation exposure to PM_10_

According to the classification-group orders categorized by the International Agency for Research on Cancer (IARC), the following classes of the studied elements are defined according to their CR: Class I elements include As and inorganic arsenic compounds, nickel compounds, and cadmium and cadmium compounds; Class 2A elements include lead compounds (inorganic); and Class 2B elements include cobalt and cobalt compounds. Copper was not classified as carcinogenic in these group orders (IARC, 2011 ▸). Because environmental pollution data are usually highly variable and subject to uncertainties of various types in addition to the uncertainties in the calculation of carcinogenic and non-carcinogenic effects due to the exposure to PM_10_, a reasonable maximum exposure concentration (EC) [the upper limit of the 95% confidence interval on the mean (95% UCL)] was used in the current exposure assessment (Gilbert, 1987 ▸; Sun *et al.*, 2014 ▸). According to the United States Environmental Protection Agency (USEPA) Human Health Evaluation Manual (Part A) (USEPA, 1989 ▸) and Supplemental Guidance for Inhalation Risk Assessment (Part F) (USEPA, 2009 ▸), the EC via inhalation of PM_10_ is estimated using the following equation,

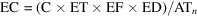

where EC is the average daily dose intakes of PM_10_-associated heavy metals for the inhalation pathway (mg kg^−1^d^−1^), C is the metal concentrations in PM_10_ (µg m^−3^), EF is the exposure frequency (350 d^−1^ year^−1^), ED is the exposure duration (six years for children and 24 years for adults), ET is the exposure time (24 hours/day) and AT_*n*_ is the average time (for carcinogens, AT_*n*_ = 70 years × 365 d year^−1^ × 24 h d^−1^). All parameters used in the calculation of EC were found in reports published by USEPA during different periods (USEPA, 2009 ▸).

The non-carcinogenic risk was evaluated using the hazard quotient (HQ) and the hazard index (HI), where HI is defined as the sum of HQs and is used to assess the cumulative risk for non-carcinogenic effects caused by more than one metal. HQs and CRs posed by toxic elements in PM_10_ via inhalation were calculated using the following equations (USEPA, 1989 ▸, 2009 ▸),

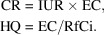

Toxicity and chemical-specific information {RfCi refers to inhalation reference concentrations (mg m^−3^), while IUR refers to inhalation unit risk [(µg m^−3^)^−1^]} were quoted from Regional Screening Levels tables for USEPA Region 9 (USEPA, 2021 ▸). The IUR values used were for As (inorganic), Cd (diet), Co, Ni (refinery dust) and Pb (acetate), and were found in the same source (USEPA, 2021 ▸). Neither the RfCi nor the IUR of Cu are available in the literature, thus Cu is not included in the calculation of carcinogenic or non-carcinogenic risk.

## 3. Results and discussion

### 3.1. Ambient concentrations of PM_10_

The average ambient-air concentration of PM_10_ in Helwan was 264 µg m^−3^, with a minimum concentration of 87.4 µg m^−3^ recorded in July and a maximum concentration of 478 µg m^−3^ recorded during May, which is clearly due to Khamaseen seasonal wind, see Table 1 ▸. The air concentration of PM_10_ in Giza ranged between 66.2 and 546.5 µg m^−3^ during late April and late October, respectively. The average concentration of PM_10_ in Giza was 214.5 µg m^−3^, as shown in Table 1 ▸. The maximum concentration could be due to resuspension of dust particles during autumn in Egypt. The average concentration of PM_10_ in Giza was lower than that in Helwan; however, the concentrations in both sites were higher in autumn and winter than in summer. The daily average limits of the Egyptian regulation (150 µg m^−3^) (EEAA, 1994 ▸) were exceeded in all samples collected from Helwan, except one sample during summer (24th July), while three samples were below the daily limits in Giza. Those three samples were collected in summer (15th and 28th April and 4th August). The daily limits of the WHO (50 µg m^−3^) were exceeded in all samples, up to ten times, when compared with the maximum concentrations in both sites (WHO, 2005 ▸). Annually, the limit of the Egyptian regulations (70 µg m^−3^) was also significantly exceeded (see Table 1 ▸). The seasonal behavior can be explained based on the mixing height of the atmosphere, which signifies the height above the earth’s surface throughout which a pollutant can be dispersed. During winter and times of surface-temperature inversions, the mixing height decreases and pollution dispersion is minimal. These conditions enhance the accumulation of the emitted PM_10_ by reducing its dispersion in air, which would raise the concentration of PM_10_, in addition to the windblown dust, especially near unpaved and desert areas. According to the wind-rose plot in Fig. 1 ▸, the sampling stations of PM_10_ are mostly affected by upwind sources in the North, NNW and NNE directions and, to a much lesser extent, by sources in the southern directions.

### 3.2. Heavy metals associated with PM_10_

In the current study, the mean concentrations of heavy metals (mean ± standard deviation) in both sites have the same descending order of Pb > Cu > Ni > Cd > Co > As, see Table 2 ▸ and Fig. S1 of the supporting information. It was observed that the concentrations of Pb, Cu, Ni and Cd in Helwan were higher than those at the Giza station, whereas the concentrations of Co and As were higher in Giza. The average concentration of Pb in the Giza samples was 36 ± 27 (11–93) ng m^−3^, while that in Helwan was 55 ± 36 (17–139) ng m^−3^. These averages are of an order of magnitude lower than the Egyptian and international air-quality-limit values for lead (500 ng m^−3^) (EEAA, 1994 ▸; WHO, 2000 ▸). According to CAIP (2000 ▸), the major sources of Pb in the GC atmosphere are lead smelters and mazut burning, while minor contribution comes from the cement industry, lead acid batteries and secondary copper processing. There was a good agreement (*R* ^2^ = 0.98) between Pb concentrations determined by ICP-MS and SR-XRF.

For comparison, Table 3 ▸ illustrates the concentrations of heavy metals associated with PM_10_ at the studied sites and at other sites worldwide. Safar & Labib (2010 ▸) assessed the lead content of PM_10_ in ten sites in the GC area during the period from 1998 to 2007 and found that the average lead concentration decreased by more than an order of magnitude during this period, *i.e.* from 3.6 µg m^−3^ in 1998 to below 0.3 µg m^−3^ in 2007. In another study, Lowenthal *et al.* (2014 ▸) measured heavy metals associated with PM_10_ collected during June and October 2010 and found that the average lead concentration in Helwan was 0.05 ± 0.03 and 0.1 ± 0.1 µg m^−3^ during June and October, respectively (see Table 3 ▸). Lead levels in Giza PM_10_ during 1993/1994, 1997/1998 and 1998/1999 recorded 1.7, 0.7 and 0.4 µg m^−3^, respectively (CAIP, 2000 ▸). This descending pattern of lead concentration is attributed to the transfer of lead smelters from Shobra Kheima to other industrial areas, in addition to the phase out of leaded gasoline since late 1997 (EEPP, 2001 ▸). Therefore, it is expected that the lead levels will continually decrease in the following years, which is confirmed by the results of the current study.

The concentrations of Ni in the Giza samples were higher than that of the unloaded blank filter in only three samples with concentrations of 7.3, 12.9 and 21.1 ng m^−3^, see Table 2 ▸. The average concentration of these three samples was 13.8 ± 7 ng m^−3^. The average concentration of Ni in Helwan was 16.6 ± 9.2 (0.2–32) ng m^−3^. This average is markedly higher than those found by Lowenthal *et al.* (2014 ▸) and Hassanien *et al.* (2009 ▸) of ∼7 ± 5 ng m^−3^ and ∼8 (6–13) ng m^−3^, respectively, given in Table 3 ▸. The average concentrations of lead and Ni were higher in Helwan than in Giza, which may be due to the presence of some mazut-fired bricks factories and a thermal power plant in the south of Helwan, in addition to the cement industry nearby. Despite Ni not having a limit value for concentration in ambient air in Egypt, its incremental health risk is given by the WHO air-quality guidelines as 3.8 × 10^−4^ (µg m^−3^)^−1^, which means that every 25 ng m^−3^ corresponds to an excess lifetime risk of 1:100 000 (Andersen, 1992 ▸; Andersen *et al.*, 1996 ▸). However, the levels of Ni concentrations were below the guide value of the EC Directive 1999/30/EC of 20 ng m^−3^ annual average (EC, 1999 ▸).

The mean concentrations of Cu in Giza and Helwan were 15.9 ± 10.2 and 21.3 ± 29.4 ng m^−3^, respectively. These concentrations are comparable with the mean concentration of Cu measured in PM_10_ samples in the western coastal area of Central Taiwan but lie within the range of Cu concentrations measured in rural and urban sites in Erbid, Jordan (Gharaibeh *et al.*, 2010 ▸), see Table 3 ▸. The mean concentrations of As, Cd and Co were below 1 ng m^−3^ in both sites. Their mean concentrations in Giza were 0.21± 0.19, 0.72 ± 0.40 and 0.39 ± 0.23 ng m^−3^, respectively, while their mean concentrations in Helwan were 0.13 ± 0.11, 0.76 ± 0.79 and 0.27 ± 0.24 ng m^−3^, respectively. It is clear from Table 3 ▸ that Cd and Co levels are also comparable with those measured in PM_10_ samples in Rome and the western coastal area of Central Taiwan, while Cd levels are significantly lower than those found in Tehran (Leili *et al.*, 2008 ▸) and Islamabad (Khan *et al.*, 2020 ▸). The average concentrations of Cd in both sites in the current study were found well below 5 ng m^−3^, which is the international limit value of Cd assigned by the WHO (WHO, 2000 ▸).

The potential source of Pb is not likely to be the vehicular emissions (because of the phase out of leaded gasoline since late 1997 and the relocation of lead smelters into terminal areas in GC) but rather may be the combustion of heavy fuel oil (mazut) used in thermal power plants north of Giza (two plants; Shobra El-Kheima and Cairo West) and two plants south of Helwan (at El-Tebbeen). The combustion of old tyres is sometimes used in bricks factories south of Helwan (at Arab Abu-Saied), which are also confirmed as sources of Ni and Co and other heavy metals (Lin *et al.*, 2015 ▸; Hsu *et al.*, 2016 ▸). The inevitable source of trace metals associated with PM_10_ is the resuspension of fine soil dust and crustal metals during autumn and seasonal wind (*e.g.* Khamaseen wind). Since there are various major arterial as well as internal routes along the GC area where cars and trucks may release and re-suspend the associated metals, traffic related emission may account for the release of Cu and Cd as traffic emissions as well as brake- and tyre-wear debris (Hsu *et al.*, 2016 ▸). Furthermore, the incineration of solid waste in some open areas in GC is another source of these metals.

The Pearson’s correlation test with 95% confidence was performed between heavy metals concentrations and the concentration of PM_10_ in both sampling stations. In Giza, there was a strong positive correlation (*p* value < 0.01) between PM_10_ and metals of Co, Ni and Pb with correlation coefficients of 0.82, 0.86 and 0.78, respectively. Moderate correlation was observed between Co–Ni (0.56) as well as Co–Cu (0.52). However, stronger correlations were found between As–Cu (0.72), Co–Pb (0.84) and Ni–Pb (0.83). In Helwan, only Co has a strong correlation (*p* value < 0.01) with PM_10_ (0.84), while moderate correlation was found between PM_10_ and Cd (0.48). A strong correlation (*p* value < 0.05) was found between As–Cu (0.72), while moderate correlations were found between Cd–Co (0.68), Cd–Ni (0.58) and Co–Cu (0.51).

The correlation between heavy metals concentrations and the concentration of PM_10_ suggests that these heavy metals are associated uniformly with particles or with the coarse portion of the PM_10_. On the other hand, the strong correlation between metals suggests a similar emission source(s) while the moderate correlation suggests contribution from different emission sources. Due to the strong correlation between PM_10_ and Pb, Ni and Co, it seems that Giza PM_10_ is more affected by the emission from legacy sites of Pb smelting as well as from the battery industry at the northern parts of GC (upwind), as those metals are tracers of Pb smelting and battery industry, see the wind rose of GC in Fig. 1 ▸.

### 3.3. XANES

#### 3.3.1. Ni *K*-edge XANES and LCF

**Figure 2:**
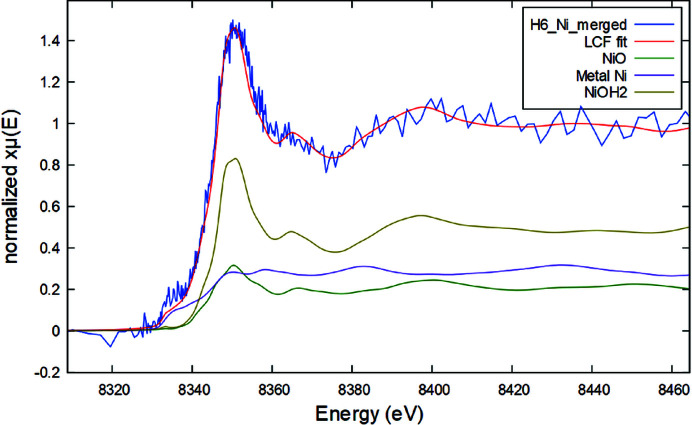
An example of a fit obtained through LCF analysis on the merged XANES spectrum of sample H6 (blue line), using 29.1% Ni metal foil (violet line), 21.6% NiO (green line) and 49.3% Ni(OH)_2_ (beige line) reference compounds.

The collected XANES data at the Ni *K*-edge (8333 eV) have a sufficient signal-to-noise ratio to perform the LCF for each PM_10_ sample; an example of a fit is represented in Fig. 2 ▸. The LCF analyses were conducted using metal Ni, NiO and Ni(OH)_2_ as standard spectra; the obtained results are summarized in Table S1 of the supporting information. By comparing the results of the two series, it is evident that generally the H samples are enriched in Ni hydroxide (especially sample H3, which is almost pure Ni hydroxide) relative to the G samples, which are composed of approximately the same proportions of metal Ni, NiO and Ni(OH)_2_. Unfortunately, the filters used to collect the PM_10_ samples are not Ni free. However, since the amount of Ni in the filter materials is constant for all the samples, we were able to determine the relative percentage of Ni compounds in the different samples under investigation.

#### 3.3.2. Pb *L*_2_-edge XANES and LCF

**Figure 3:**
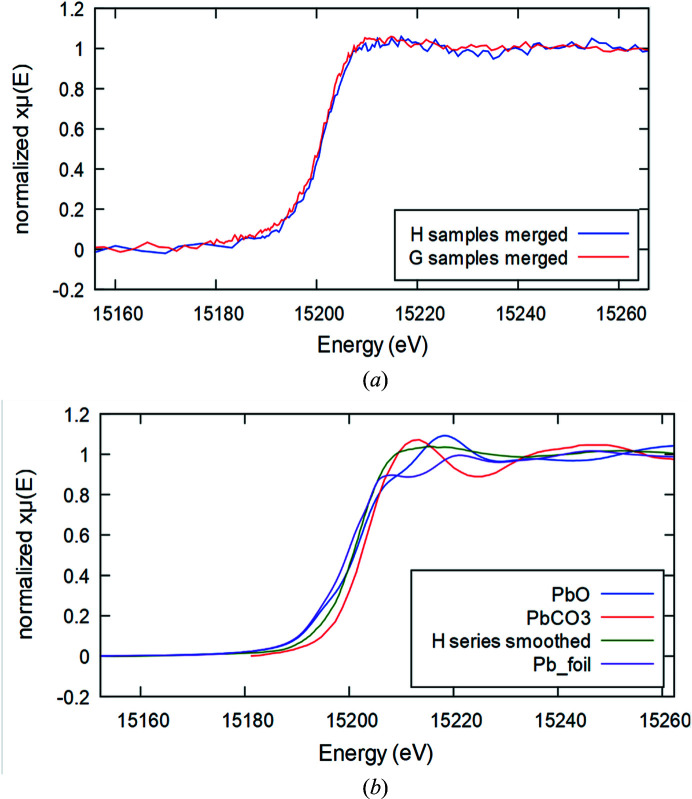
(*a*) Comparison between the Pb *L*_2_-edge normalized spectra representative of the H and G sample series. (*b*) Reference spectra of Pb-bearing compounds [Pb metal foil, PbO and PbCO_3_ taken from Lanzirotti *et al.* (2014 ▸)] compared with the representative spectrum of the H series (with soft smoothing for better visualization).

The XANES spectra collected at the Pb *L*_2_-edge (15 200 eV) have a considerably low signal-to-noise ratio due to the low amount of Pb within PM_10_ when compared with matrix elements, especially in the G samples. This has prevented us from performing LCF analysis. However, from merging all the spectra of the G series and all that of the H series, some information can be obtained. First, Pb is very likely in similar chemical environments among the two series, G and H, as the two representative spectra have the same edge position and similar features [Fig. 3 ▸(*a*)].

Furthermore, from comparing these representative spectra with those of Pb reference compounds, an indication about the Pb oxidation state can be obtained; indeed, the average spectrum of the H samples falls between reference spectra of metal Pb and Pb^2+^ [Fig. 3 ▸(*b*)].

**Figure 4:**
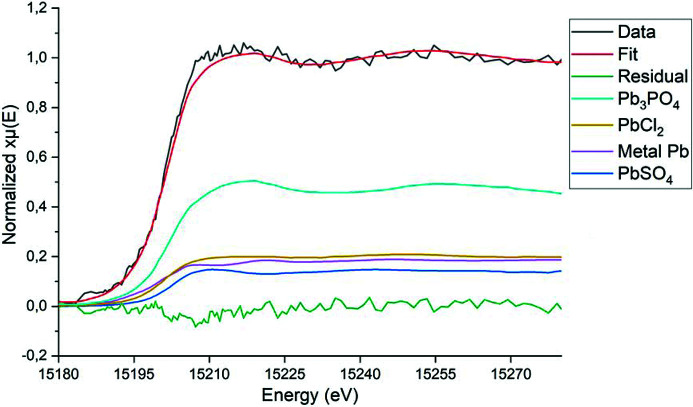
A fit obtained through LCF analysis of the Pb *L*_2_-edge merged spectrum of the H samples, using Pb metal foil, PbCl_2_, PbSO_4_ and Pb_3_PO_4_ spectra as standard references [last three taken from Lanzirotti *et al.* (2014 ▸), extracted using the free online tool *WebPlotDigitizer* (Rohatgi, 2014 ▸)].

In addition, a tentative LCF analysis was conducted on the merged spectrum of the H series sample. The result (Fig. 4 ▸) suggests the presence of PbCl_2_, PbSO_4_, metal Pb and Pb_3_PO_4_ (20, 15, 19 and 46%, respectively, despite being merely indicative as affected by high sigmas of ±10). Among the Pb^2+^ species, sulfates are a secondary pollutant and are usually formed as a result of reactions of metals with sulfur oxides in the atmosphere. Accordingly, the presence of sulfate is evidence of combustion processes *e.g.* thermal power plants and industrial facilities. The presence of chloride could be attributed to biomass burning as well as the uncontrolled incineration of solid waste. Phosphates and carbonates may have originated from terrestrial sources, fertilizers and cement industries.

In a study published by Shaltout *et al.* (2019 ▸) on the lead speciation in PM_2.5_ from two sites (residential and industrial) in GC, Egypt, and one site in Zarqa, Jordan, during winter 2015, they found that the most dominant Pb species was the divalent lead Pb(II) in the residential area of Cairo as well as in the urban area of Zarqa. The chemical forms of Pb(II) were chloride (PbCl_2_) and relatively similar amounts of lead oxalate (PbC_2_O_4_) and lead hydroxyl carbonate Pb_3_(CO_3_)_2_(OH)_2_. They attributed the presence of Pb(II) compounds to natural and anthropogenic sources, such as soil, street dust, metal processing of material containing lead, lead-based paints, and accumulated lead in the old exhaust systems.

### 3.4. Risk of exposure to PM_10_ in Cairo

For carcinogens, the acceptable risk range is between 1 × 10^−6^ and 1 × 10^−4^ using USEPA’s risk management. An HQ and/or HI below one indicates that there is no significant risk of non-carcinogenic effects. Consequently, their values above these defined risk limits indicate that there is a possibility of health effects occurring, with a probability that tends to increase as the value of HQ/HI increases (USEPA, 1989 ▸). Carcinogenic and non-carcinogenic risks from Ni inhalation were excluded from the calculation because of the high blank concentrations of Ni compared with that of the particulates themselves. Therefore, Ni concentrations resulted from blank subtraction may result in erroneous risk values.

Carcinogenic and non-carcinogenic risks from toxic elements in PM_10_ via inhalation exposure are given in Table S2. The CRs via inhalation exposure of As, Cd, Co and Pb to children and adults were below the acceptable level (1 × 10^−4^), and were found to decrease in the order Co > Pb > As > Cd in Giza while decreasing in the order Pb > Co > Cd > As in Helwan, for both age categories. Furthermore, the integrated risks from the inhalation of these elements were also below the acceptable levels for adults and children of Giza (5.8 × 10^−6^ and 1.4 × 10^−6^, respectively) and Helwan (6.0 × 10^−6^ and 1.5 × 10^−6^, respectively). The oxidation state and the chemical form of the metal are of considerable importance from the view of CR. From the results of XANES, Pb was found as Pb^2+^ and, to a much lesser extent, as Pb^0^ metal. Pb^2+^ is expected to be more bioavailable than metallic Pb; therefore, it is more absorbed by living tissues and consequently poses more risk. Furthermore, the different chemical forms of the same oxidation state of a metal may also have different toxicities. For example, Pb(II) acetate is about seven times more toxic than Pb(II) phosphate (USEPA, 2021 ▸). The non-carcinogenic risk from the inhalation of the measured toxic metals has the same HQ values for both adult and children and was below the safe limit (HQ = 1) for every single element. The HI value from the inhalation of those toxic elements in Giza and Helwan was also below the safe level (= 1), with a slight increase in Helwan (3 × 10^−1^) compared with that of Giza (2.8 × 10^−1^). It is clear from Table S2 that the highest non-carcinogenic risk in Giza is due to the inhalation of Co, whereas that in Helwan is due to the inhalation of Cd, given that Pb is not included in the non-carcinogenic risk because its RfCi are not available in the literature.

### 3.5. Limitations of the study

Although the glass fiber filter is characterized by its superior collection efficiency of more than 98% of the PM_10_, one of its significant limitations is its high content of Ni relative to the concentrations associated with the PM_10_ sampled onto it. This issue is difficult to handle by blank subtraction, especially using XRF. For that reason, Ni was excluded from the calculation of risk assessment. For toxicological assessment, carcinogenic and non-carcinogenic health risks are evaluated based on a number of assumptions, which may introduce uncertainties and limitations into the risk assessment. Some of these uncertainties come from toxicity parameters used in the risk from inhalation. For example, the IUR is available for both lead acetate and lead phosphate but the use of lead acetate values result in a higher CR. Therefore, since compound-specific values of toxicity parameters are not available, conservative values of IUR were used in order to reduce the probability of underestimation of the assessed health risks.

## 4. Conclusions

In this work we have combined synchrotron-based techniques (XANES and XRF) along with ICP-MS to perform the chemical speciation of lead and nickel and to assess the concentration of some heavy metals associated with PM_10_ as toxic elements with considerable public health impacts. The concentrations of Pb, Cu, Ni and Cd in Helwan were higher than those in Giza, whereas the concentrations of Co and As were higher in Giza. The major sources of Pb in the GC atmosphere are lead smelters and heavy fuel-oil (mazut) combustion, lead acid batteries and secondary copper processing, with minor contribution from the cement industry. The concentrations of Ni in the Giza samples were lower than that of the blank filter in most of the samples. However, Ni concentrations were below the annual guidance value of the EC Directive 1999/30/EC of 20 ng m^−3^, in both sites. The XRF results of Pb showed a good agreement with the ICP-MS results, but the XRF results of Ni were not meaningful because of the high Ni content of the blank filters. The average spectrum of the H samples suggests that lead was found mostly as Pb^2+^ and, to a lesser extent, as metal Pb. The potential Pb^2+^ chemical forms according to the comparison with the XANES spectra of the model standard compounds could be PbCO_3_, PbSO_4_, PbCl_2,_ Pb_3_(PO4)_2_ and PbO. With respect to the risk via inhalation exposure, all the studied elements resulted in carcinogenic and non-carcinogenic risk for adults and children that are below the acceptable levels in Giza and Helwan. Finally, the integration between ICP-MS and synchrotron XANES in this study provides a complete view of concentrations of heavy metals associated with PM_10_ and the speciation of Pb and Ni in PM over GC. The obtained results will help decision-makers evaluate pollution control strategies in order to cope with national and international regulations and consequently protect public health.

## Supplementary Material

Tables S1 and S2; Figure S1. DOI: 10.1107/S1600577522003058/rv5161sup1.pdf

## Figures and Tables

**Table 1:** Concentrations of PM_10_ in ambient air over Helwan University and Giza Square sampling sites in 2018, with indication of the temperature range and relative humidity range

Sampling date	Helwan PM_10_ conc. (µg m^−3^)	*T* (°C) (min.–max.)	Rh% (min.–max.)	Sampling date	Giza PM_10_ conc. (µg m^−3^)	*T* (°C) (min.–max.)	Rh% (min.–max.)
29th January (H9)	213.60	7–17	48–68				
27th February (H7)	272.89	13–25	18–65				
20th February (H8)	203.12	9–21	22–71				
				28th April (G1)	66.15	18–35	16–78
30th April (H1)	383.74	21–36	7–34	15th April (G2)	94.60	15–29	17–83
8th May (H6)	478.71	20–33	19–60	19th May (G3)	152.36	22–39	13–53
26th June (H5)	157.20	23–37	16–61				
24th July (H4)	87.37	22–35	23–80				
				4th August (G5)	98.46	24–33	38–79
				18th August (G6)	189.69	24–34	30–83
23rd October (H3)	245.12	17–28	34–83	7th October (G8)	209.62	17–25	30–59
30th October (H2)	450.69	16–27	32–76	27th October (G9)	546.46	16–26	32–88
25th December (H10)	146.72	9–18	14–28	22nd December (G4)	289.45	10–18	40–94
				29th December (G7)	284.05	10–17	24–51
							
Average	263.92				214.54		
Minimum	87.37				66.15		
Maximum	478.71				546.46		
Standard deviation	132.6				147.9		
WHO daily limits (2005 ▸)	50				50		
Egyptian annual limits	70				70		

**Table 2:** Concentrations of toxic heavy metals (ng m^−3^) associated with PM_10_ (µg m^−3^) at the Giza and Helwan stations, as well as the detection limit (DL) of the ICP-MS (ng l^−1^)

	PM_10_ (µg m^−3^)	As (ng m^−3^)	Cd (ng m^−3^)	Co (ng m^−3^)	Cu (ng m^−3^)	Ni (ng m^−3^)	Pb (ng m^−3^)
Giza							
G1	66.15	0.124 ± 0.002	0.81 ± 0.06	0.253 ± 0.004	18.33 ± 0.57	–	22.38 ± 0.57
G2	94.6	0.028 ± 0.001	0.4 ± 0.03	0.108 ± 0.003	4.74 ± 0.15	–	10.29 ± 0.26
G3	152.4	0.246 ± 0.004	1.05 ± 0.07	0.515 ± 0.008	16.39 ± 0.47	7.26 ± 0.13	28.86 ± 0.71
G4	289.5	0.325 ± 0.004	1.56 ± 0.12	0.258 ± 0.005	12.04 ± 0.37	–	24.85 ± 0.69
G5	98.5	0.040 ± 0.001	0.26 ± 0.02	0.144 ± 0.002	5.79 ± 0.17	12.9 ± 0.23	14.44 ± 0.37
G6	189.7	0.081 ± 0.001	0.45 ± 0.03	0.411 ± 0.007	5.31 ± 0.15	–	32.83 ± 0.81
G7	284.1	0.660 ± 0.011	0.62 ± 0.05	0.496 ± 0.007	31.92 ± 0.90	–	22.14 ± 0.54
G8	209.6	0.185 ± 0.003	0.53 ± 0.04	0.464 ± 0.009	30.05 ± 0.87	–	66.82 ± 1.61
G9	546.5	0.223 ± 0.004	0.82 ± 0.06	0.844 ± 0.014	18.71 ± 0.54	21.1 ± 0.41	92.73 ± 2.26
Helwan							
H1	338.7	0.273 ± 0.003	2.86 ± 0.20	0.712 ± 0.029	20.64 ± 0.97	31.99 ± 1.42	74.41 ± 1.88
H2	450.7	0.143 ± 0.002	0.57 ± 0.04	0.683 ± 0.011	75.54 ± 2.25	15.2 ± 0.28	78.59 ± 1.93
H3	245.1	0.390 ± 0.007	0.46 ± 0.03	0.213 ± 0.004	77.07 ± 2.17	21.95 ± 0.41	38.99 ± 0.94
H4	87.4	0.030 ± 0.001	0.21 ± 0.02	0.080 ± 0.001	3.61 ± 0.11	15.93 ± 0.30	16.48 ± 0.40
H5	157.2	0.080 ± 0.001	0.34 ± 0.02	0.161 ± 0.004	8.08 ± 0.24	12.53 ± 0.27	20.24 ± 0.49
H6	478.7	0.108 ± 0.002	0.8 ± 0.06	0.394 ± 0.017	9.68 ± 0.40	13.36 ± 0.54	67.1 ± 1.65
H7	272.9	0.042 ± 0.001	1.0 ± 0.07	0.190 ± 0.003	6.31 ± 0.19	26.06 ± 0.49	36.85 ± 0.89
H8	203.1	0.090 ± 0.002	0.91 ± 0.06	0.126 ± 0.003	6.48 ± 0.21	–	46.26 ± 1.11
H9	213.6	0.084 ± 0.002	0.26 ± 0.02	0.108 ± 0.002	3.79 ± 0.12	12.07 ± 0.26	30.13 ± 0.73
H10	146.7	0.048 ± 0.001	0.2 ± 0.01	0.041 ± 0.001	2.06 ± 0.06		138.02 ± 3.91
							
Blank filter	–	0.05	0.17	0.05	0.3	143.5	5.1
DL (ng l^−1^)		2.9	8.5	2.6	2.9	176	7.9

**Table 3:** Concentrations of heavy metals (ng m^−3^) associated with PM_10_ in the studied samples and other areas worldwide The literature data were not presented in a uniform way, sometimes with errors sometimes with ranges.

	As (ng m^−3^)	Cd (ng m^−3^)	Co (ng m^−3^)	Cu (ng m^−3^)	Ni (ng m^−3^)	Pb (ng m^−3^)	Reference
Giza, Egypt	0.21 ± 0.19	0.72 ± 0.40	0.39 ± 0.23	15.92 ± 10.15	13.75 ± 6.96	35.04 ± 27.04	Current study
Helwan, Egypt	0.13 ± 0.11	0.76 ± 0.79	0.27 ± 0.24	21.33 ± 29.43	16.59 ± 9.18	54.71 ± 36.43	Current study
Helwan, Egypt			17 (13–27)	57 (49–64)	8 (6–13)		Hassanien *et al.* (2009 ▸)
Helwan, Egypt					7 ± 5	50 ± 30	Lowenthal *et al.* (2014 ▸)
Algris, Algeria		21.2	37.7	102.8	42.4	299.3	Oucher *et al.* (2015 ▸)
Western coastal area (Taiwan)	3.39 ± 2.42	0.704 ± 0.660	0.531 ± 0.473	15.7 ± 10.0	9.84 ± 17.81	21.2 ± 20.7	Hsu *et al.* (2016 ▸)
Rome, Italy	1.35 ± 0.89	0.526 ± 0.253	0.379 ± 0.18		4.54 ± 2.34	92 ± 47.8	Avino *et al.* (2008 ▸)
Erbid, Jordan (urban site)				117 (8–316)	8 (2–17)	111 (15–221)	Gharaibeh *et al.* (2010 ▸)
(rural site)				55 (4–145)	5 (1–11)	21 (4–40)	Gharaibeh *et al.* (2010 ▸)
Tehran, Iran		6.87 ± 2.22				132.53 ± 109.34	Leili *et al.* (2008 ▸)
Islamabad, Pakistan		10		170	10	350	Khan *et al.* (2020 ▸)
Air-quality-limit values		5†			20‡	500† §	WHO (2000 ▸), EC (1999 ▸), EEAA (1994 ▸)

† WHO (2000 ▸).

‡ European Council Directive 1999/30/EC (EC, 1999 ▸).

§ Egyptian law of environment (EEAA, 1994 ▸).
