# Supplementary material for: Heavy metals concentrations and speciation of Pb and Ni in airborne particulate matter over two residential sites in Greater Cairo – reflection from synchrotron radiation
Source: J Synchrotron Radiat. 2022 Apr 22;29(Pt 3):765–74. doi: 10.1107/S1600577522003058 (PMC9070700; doi:10.1107/S1600577522003058)
Supplement: Supplementary file 1 [file s-29-00765-sup1.pdf]

JOURNAL OF SYNCHROTRON RADIATION

Volume 29 (2022)

Supporting information for article:

Heavy metals concentrations and speciation of Pb and Ni in airborne particulate matter over two residential sites in Greater Cairo; reflection from synchrotron radiation

Mohamed H. E. Monged, N. G. Imam, Giuliana Aquilanti, Simone Pollastri, A. M. Rashad and János Osán

Table S1 Results of the LCF analysis on G and H samples (the reported quantities are percentages).

**Sample**	**Metal Ni (%)**	**NiO (%)**	**$Ni(OH)_2$ (%)**	**Sum**
**G1**	56.8 (4)	16.2 (8)	27.0 (7)	100
**G2**	49.8 (3)	25.2 (6)	25.1 (8)	100
**G3**	26.4 (2)	13.1 (6)	60.5 (6)	100
**G4**	44.7 (3)	21.5 (5)	33.8 (8)	100
**G5**	24.4 (1)	30.4 (7)	45.2 (8)	100
**G6**	32.3 (2)	26.8 (4)	40.9 (4)	100
**G7**	46.9 (3)	26.4 (6)	26.7 (7)	100
**G8**	38.8 (3)	23.0 (9)	38.3 (7)	100
**G9**	26.2 (1)	28.6 (5)	45.1 (5)	100
**G mean values**	38.5	23.5	38.1	100
**H1**	25.3 (4)	19.6 (6)	55.1 (8)	100
**H2**	14.3 (2)	25.7 (7)	60.0 (9)	100

**H3**	0.0 (1)	0.7 (5)	99.3 (7)	100
**H4**	22.8 (3)	12.8 (6)	64.4 (6)	100
**H5**	15.2 (4)	18.3 (9)	66.5 (5)	100
**H6**	29.1 (4)	21.6 (6)	49.3 (8)	100
**H7**	12.5 (3)	7.5 (7)	80.0 (6)	100
**H8**	19.8 (2)	17.4 (8)	62.7 (10)	100
**H9**	28.7 (3)	13.5 (7)	57.8 (6)	100
**H10**	23.3 (5)	8.9 (6)	67.9 (5)	100
**H mean values**	19.1	14.6	66.3	100

Table S2 Carcinogenic and non-carcinogenic risks from toxic elements through inhalation of PM_{10}.

	**95% UCL Conc**	**Carcinogenic inhalation exposure**		**Non-carcinogenic inhalation exposure**		**IUR**	**RfCi**	**Carcinogenic risk**		**Non-carcinogenic Risk**	
**Giza**	(ng/m^3)	EC-Adult	EC-Child	EC-Adult	EC-Child	(μg/m^3)$^{-1}$	(mg/m^3)	Adult	Child	Adult	Child
**As**	0.70	2.29E-04	5.74E-05	6.69E-04	6.69E-04	4.30E-03	1.50E-05	9.86E-07	2.47E-07	4.46E-02	4.46E-02
**Cd**	1.17	3.84E-04	9.59E-05	1.12E-03	1.12E-03	1.80E-03	1.00E-05	6.90E-07	1.73E-07	1.12E-01	1.12E-01

**Co**	0.76	2.50E-04	6.25E-05	7.29E-04	7.29E-04	9.00E-03	6.00E-06	2.25E-06	5.62E-07	1.22E-01	1.22E-01
**Pb**	70.10	2.30E-02	5.76E-03	6.72E-02	6.72E-02	8.00E-05	-	1.84E-06	4.61E-07	-	-
**Sum**								5.77E-06	1.44E-06	2.79E-01	2.79E-01
**Helwan**											
**As**	0.28	9.13E-05	2.28E-05	2.66E-04	2.66E-04	4.30E-03	1.50E-05	3.92E-07	9.81E-08	1.77E-02	1.77E-02
**Cd**	1.70	5.60E-04	1.40E-04	1.63E-03	1.63E-03	1.80E-03	1.00E-05	1.01E-06	2.52E-07	1.63E-01	1.63E-01
**Co**	0.71	2.35E-04	5.87E-05	6.85E-04	6.85E-04	9.00E-03	6.00E-06	2.11E-06	5.29E-07	1.14E-01	1.14E-01
**Pb**	96.7	1.05E-02	2.63E-03	3.07E-02	3.07E-02	8.00E-05	-	2.54E-06	6.36E-07	-	-
**Sum**								6.05E-06	1.52E-06	2.95E-01	2.95E-01

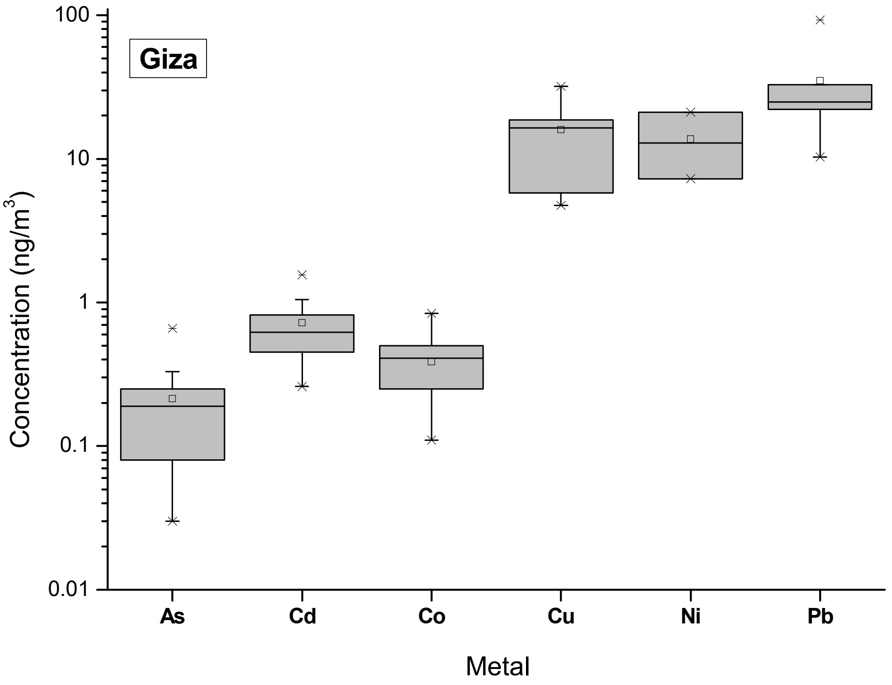

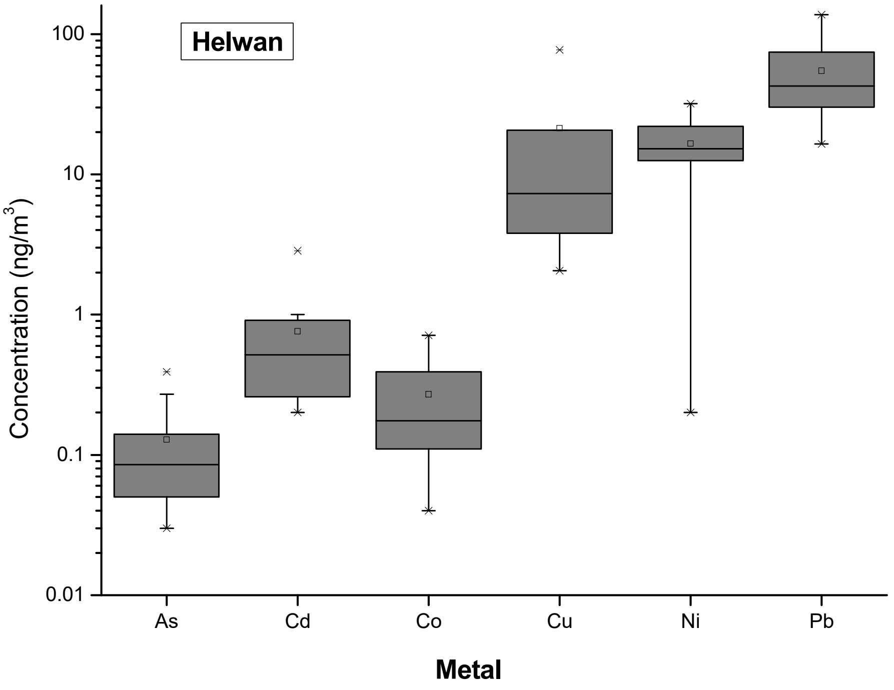

Figure S1 The concentrations of heavy metals (ng/m^3) associated with PM_{10} at Giza (up) and Helwan (down).
